# Minimally-invasive bedside catheter haematoma aspiration followed by local thrombolysis in spontaneous supratentorial intracerebral haemorrhage: a retrospective single-center study

**DOI:** 10.3389/fneur.2023.1188717

**Published:** 2023-06-05

**Authors:** Maren Hieber, Johann Lambeck, Amjad Halaby, Roland Roelz, Theo Demerath, Wolf-Dirk Niesen, Jürgen Bardutzky

**Affiliations:** ^1^Department of Neurology and Neurophysiology, Medical Center – University of Freiburg, Faculty of Medicine, University of Freiburg, Freiburg, Germany; ^2^Department of Neurosurgery, Medical Center – University of Freiburg, Faculty of Medicine, University of Freiburg, Freiburg, Germany; ^3^Department of Neuroradiology, Medical Center – University of Freiburg, Faculty of Medicine, University of Freiburg, Freiburg, Germany

**Keywords:** spontaneous intracerebral haemorrhage, minimally-invasive surgery, urokinase, local thrombolysis, perihemorrhagic edema

## Abstract

**Background and purpose:**

The role of surgery in the treatment of intracerebral haemorrhage (ICH) remains controversial. Whereas open surgery has failed to show any clinical benefit, recent studies have suggested that minimal invasive procedures can indeed be beneficial, especially when they are applied at an early time point. This retrospective study therefore evaluated the feasibility of a free-hand bedside catheter technique with subsequent local lysis for early haematoma evacuation in patients with spontaneous supratentorial ICH.

**Methods:**

Patients with spontaneous supratentorial haemorrhage of a volume of >30 mL who were treated with bedside catheter haematoma evacuation were identified from our institutional database. The entry point and evacuation trajectory of the catheter were based on a 3D-reconstructed CT scan. The catheter was inserted bedside into the core of the haematoma, and urokinase (5,000 IE) was administered every 6 h for a maximum of 4 days. Evolution of haematoma volume, perihaemorrhagic edema, midline-shift, adverse events and functional outcome were analyzed.

**Results:**

A total of 110 patients with a median initial haematoma volume of 60.6 mL were analyzed. Haematoma volume decreased to 46.1 mL immediately after catheter placement and initial aspiration (with a median time to treatment of 9 h after ictus), and to 21.0 mL at the end of urokinase treatment. Perihaemorrhagic edema decreased significantly from 45.0 mL to 38.9 mL and midline-shift from 6.0 mm to 2.0 mm. The median NIHSS score improved from 18 on admission to 10 at discharge, and the median mRS at discharge was 4; the latter was even lower in patients who reached a target volume ≤ 15 mL at the end of local lysis. The in-hospital mortality rate was 8.2%, and catheter/local lysis-associated complications occurred in 5.5% of patients.

**Conclusion:**

Bedside catheter aspiration with subsequent urokinase irrigation is a safe and feasible procedure for treating spontaneous supratentorial ICH, and can immediately reduce the mass effects of haemorrhage. Additional controlled studies that assess the long-term outcome and generalizability of our findings are therefore warranted.

**Clinical trial registration:**

[www.drks.de], identifier [DRKS00007908].

## 1. Introduction

Intracerebral haemorrhage (ICH) accounts for approximately 20% of all strokes and represents the most detrimental subtype of stroke, with a 30-day mortality rate of around 40% (1).

There are no proven beneficial therapies for ICH to date (2), other than specialized care on a stroke unit and early blood pressure control. Moreover, the role of surgery remains controversial. Although surgical removal of clots can potentially reduce the direct mass effect of the haematoma on the surrounding brain tissue and hence prevent perihaematomal edema (PHE) and reduce ensuing cellular toxicity from blood products (3–5), such beneficial effects could not be validated in the two large randomized STICH (*Surgical treatment for intracerebral hemorrhage*) trials (6, 7). However, despite being open to the inclusion of all surgical procedures, the vast majority of surgical procedures analysed in these trials comprised of craniotomy (75 and 99% in STICH and STICH II, respectively).

To circumvent the potentially adverse effects of open surgery, several techniques using minimal invasive surgery (MIS) have been tested for clot removal, yielding encouraging results (8, 9). The most recent MISTIE trials (*Minimally Invasive Surgery Plus Alteplase for Intracerebral Hemorrhage Evacuation*) compared the efficacy of image-guided MIS with supplementary thrombolytic irrigation to standard medical care. However, despite a significant decrease in haematoma volume, MISTIE failed to demonstrate an overall benefit in functional outcome. Of note, surgical treatment in the MISTIE III trial was initiated 58 h post-ictus, but subgroup analysis showed a trend toward MIS being superior to standard medical care when treatment started ≤36 h after ictus (10). A recent meta-analysis of 21 studies showed that the chance of a good functional outcome was 40% higher after any type of surgery, and 47% higher after MIS when compared to medical therapy. Furthermore, a specific meta-analysis of craniotomy studies revealed no significant effect on functional outcome (RR 1.44, CI 0.69–2.93). In general, the sooner intervention is performed after symptom onset, the more effective it appears to be (11). Based on this, the current American and German guidelines state that image-guided minimally invasive haematoma evacuation (without further specific definition of the procedure) can potentially improve both mortality and outcome when spontaneous supratentorial ICH volumes exceed 30 mL (12, 13).

We recently reported on the feasibility and safety of a bedside, free-hand, minimally invasive catheter technique in specific ICH subgroups, including those associated with cerebral amyloid angiopathy (14), the use of vitamin K antagonists (15), or intravenous thrombolysis in ischaemic stroke (16).

In contrast to most of the existing MIS techniques, this approach offers the advantage of immediate feasibility, without the need for specific stereotactic or image-guided procedures. Furthermore, it is not restricted by factors such as operating room availability, anesthesia requirements and radiological resources. Taken together, these advantages can help shorten the delay between the event and haematoma evacuation.

We therefore performed a retrospective study on the feasibility and efficacy of this free-hand, bedside, minimally invasive technique in patients with large spontaneous supratentorial ICH, who represent a major subgroup of ICH patients.

## 2. Methods

### 2.1. Patients selection

Patients with spontaneous supratentorial ICH who were admitted to the neurological department of a tertiary care center between 2011 and 2021 were retrospectively identified in a prospective institutional database.

Patients were treated with free-hand bedside MIS if the following criteria were fulfilled:

- spontaneous lobar or ganglionic supratentorial ICH with a haematoma volume > 30 mL;
- exclusion of an underlying vascular malformation in CT- or MR-angiography;
- reduced level of consciousness due to ICH, defined by the Glasgow Coma Scale (GCS) <14;
- normal coagulation values, defined by International Normalized Ratio (INR) <1.3, partial thromboplastin time (PTT) <35 s, platelet count >100.000/μl.

Patients with early withdrawal of care (within 24 h of admission) were excluded from analysis. Patients with ICH related to probable cerebral amyloid angiopathy (*n* = 21), anticoagulation (*n* = 32), or systemic thrombolysis with recombinant tissue plasminogen activator for ischaemic stroke (*n* = 12), were also excluded from the present study, but analyzed and described elsewhere (14–16).

The MIS procedure was typically performed within 24 h of symptom onset. Patients were excluded if the bedside MIS procedure was performed outside a 48-h time window. In addition, patients were only included in the analysis if they had at least one CT scan before, and one immediately after, drainage placement. This study was approved by our local ethics committee (IRB number: 161/19; clinical trial registration ID: DRKS00007908) and informed consent was obtained from every patient or the next of kin.

### 2.2. Medical treatment

All patients received medical treatment according to local institutional guidelines as well as the existing European Stroke Initiative guidelines for monitoring and treating ICH (17, 18), which includes the immediate reduction of systolic blood pressure to <140–160 mmHg.

According to the institutional protocol, patients with clinical and radiological signs of incipient herniation due to the mass effect of the ICH were considered for open surgery as a life-saving option.

### 2.3. Catheter placement and local thrombolysis

**Figure 1:**
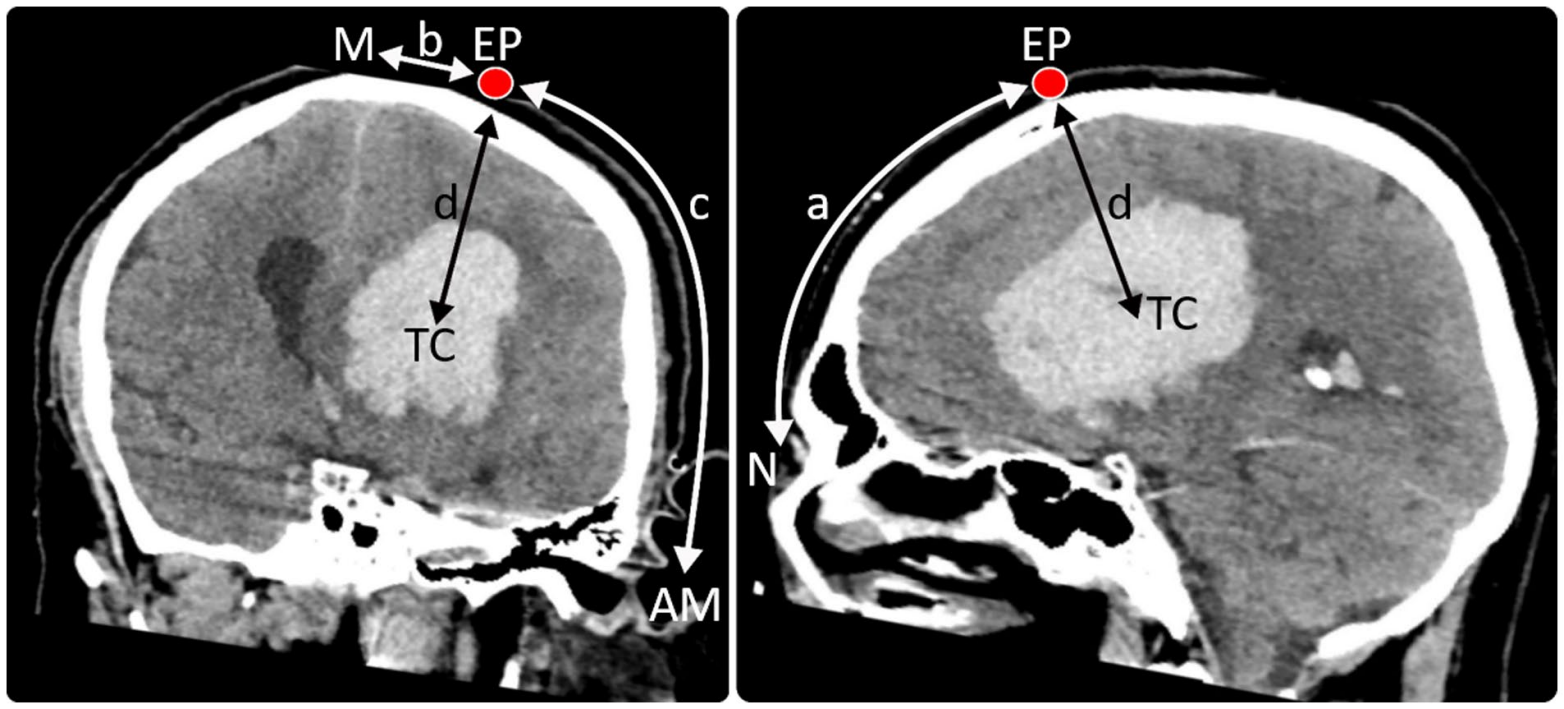
Entry point (EP) and trajectory of the catheter were planned using a 3D reconstructed CT scan. The modified technique described by Seeger, 1980 (*) was used to determine the distances of the entry point (EP) from the nasion (N) (distance a), midline (M) (distance b), and acoustic meatus (AM) (distance c) as well as the distance between the skin level to the target position of the tip of the catheter (TC) within the haematoma (distance d) in the 3D image. These coordinates were then transferred to the patient’s head. (*) Microsurgery of the Brain: Anatomical and Technical Principles. Author: Prof. Dr. med. Wolfgang Seeger (auth.); Publisher: Springer Vienna, Year: 1980; pp. 46–53.

Based on the extent of the ICH in axial, sagittal and transversal planes in a 3D-reconstructed cerebral CT the most favorable catheter trajectory for sparing critical intracerebral structures was determined. From here, the CT scan was used to determine: (i) the location for the creation of the mini burr hole, including its distance from the midline, the nasion and the acoustic meatus as well as (ii) the distance between the skin level to the target position of the tip of the catheter within the haematoma (see Figure 1).

In patient’s supine position the midline was determined by calculating the midpoint of the circumference from one acoustic meatus to the other. The coordinates for the mini burr hole determined as described above were transferred to the patient’s head, hair was shaved in a respective area of 6x6cm, and the skin was sanitized with ethanol/isopropyl alcohol. Local anesthesia of skin and periosteum was achieved by infiltration with 50 mg mepivacaine, and propofol was used for conscious sedation (median dose 95 mg, range 60 to 170 mg).

An electric twist drill was then used to make a 3.5 mm burr hole in the skull at the marked position and to perforate the dura. Subsequently, a scaled external ventricular catheter (10 French, without antibiotic or silver impregnation, Spiegelberg, Hamburg, Germany) was inserted at the calculated angle and depth. In general, frontal trajectories were used for patients with deep ICH occupying the anterior basal ganglia, with an entry point at the forehead, while posterior trajectories were used for patients with deep ICH occupying the posterior basal ganglia or thalamus, with an entry point at the parieto-occipital skull. The trajectorial entry point in lobar ICH patients was the superficial area closest to the haematoma.

After catheter placement, mild aspiration by connecting a 10–20 mL hand-held syringe to the catheter was performed for immediate blood drainage. For this, the piston of the syringe was carefully retracted until the first sign of resistance, or – in cases of aspiration volume exceeding 20 mL – a new syringe was connected and careful aspiration was repeated until the first sign of resistance. Post aspiration, the syringe was removed, the catheter was attached to the skin, and a sterile drainage system was connected. The collecting chamber of the drainage system was set at minus 30 − 40 cm H_2_O below the foramen of Monro to allow for gravitational drainage of the clot.

A follow-up cranial CT was performed to verify catheter position and to define residual haematoma volume after aspiration. Catheter position was considered to be satisfactory when the fenestrated distal segment was fully engaged with the blood clot. If the follow-up CT revealed an unsatisfactory catheter position, catheter placement was re-attempted, either by simple retraction (if the tip the catheter had been placed too deeply) or by a completely new placement process, where necessary.

### 2.4. Local thrombolysis

In cases where one-step catheter aspiration did not reduce the haematoma volume to ≤15 mL, urokinase (5,000 IE, 1 mL) was injected 3 h after catheter placement into the drainage system, and the system was then flushed with 2 mL of 0.9% saline. The drain was clamped for 30 min and subsequently re-opened to allow drainage of the lysed clot. Local thrombolysis (each 5,000 IE urokinase, 1 mL) was repeated every 6 h either until haematoma volume decreased to ≤15 mL, or for a maximum of 4 days.

### 2.5. Assessment of ICH volume, perihaematomal edema (PHE) and midline shift

Neuroimaging was performed on a 4th-generation CT scanner (Somatom Definition AS, Siemens Healthcare, Erlangen, Germany). Each CT scan consisted of 42–45 slices (each 3 mm) for the entire brain. CT images were acquired using the orbito-meatal plane. Absolute volumes (ml) of ICH and PHE were determined by means of a validated semi-automatic volumetric algorithm, as previously described (19). Midline shift (mm) was determined by measuring the distance between the centre of the third ventricle and a designated midline drawn between the anterior and posterior attachments of the falx to the inner table of the skull.

ICH volume, PHE volume and midline shift were determined in each patient by CT (i) at admission, (ii) immediately after catheter placement, and (iii) after thrombolysis was completed (day 3 to day 4).

### 2.6. Clinical and radiological endpoints

The National Institutes of Health Stroke Scale (NIHSS; scores ranging from 0 to 42, with higher scores indicating a greater deficit) and GCS scores were obtained from medical records, both on admission and at discharge. The Modified Rankin Scale (mRS) score at discharge was also retrieved.

Evolution of haematoma, edema volume and midline shift over time were analyzed as primary imaging variables. Secondary haematoma expansion after catheter placement/thrombolysis and the occurrence of catheter infections were assessed as safety parameters.

### 2.7. Statistics

Statistical analyses were performed using the IBM® SPSS® Statistics 21 software package (IBM-Corporation, Armonk, NY) and R 2.12.0.^1^ The significance level was set at *α* = 0.05 and statistical tests were 2-sided. We used the Kolmogorov–Smirnov test to determine the distribution of data. Since the data turned out to be non-normally distributed, data were presented as median and interquartile range (IQR), and were compared using the non-parametric Wilcoxon test (paired data) or the Mann–Whitney *U* test (unpaired data).

## 3. Results

### 3.1. Patients` characteristics

**Figure 2:**
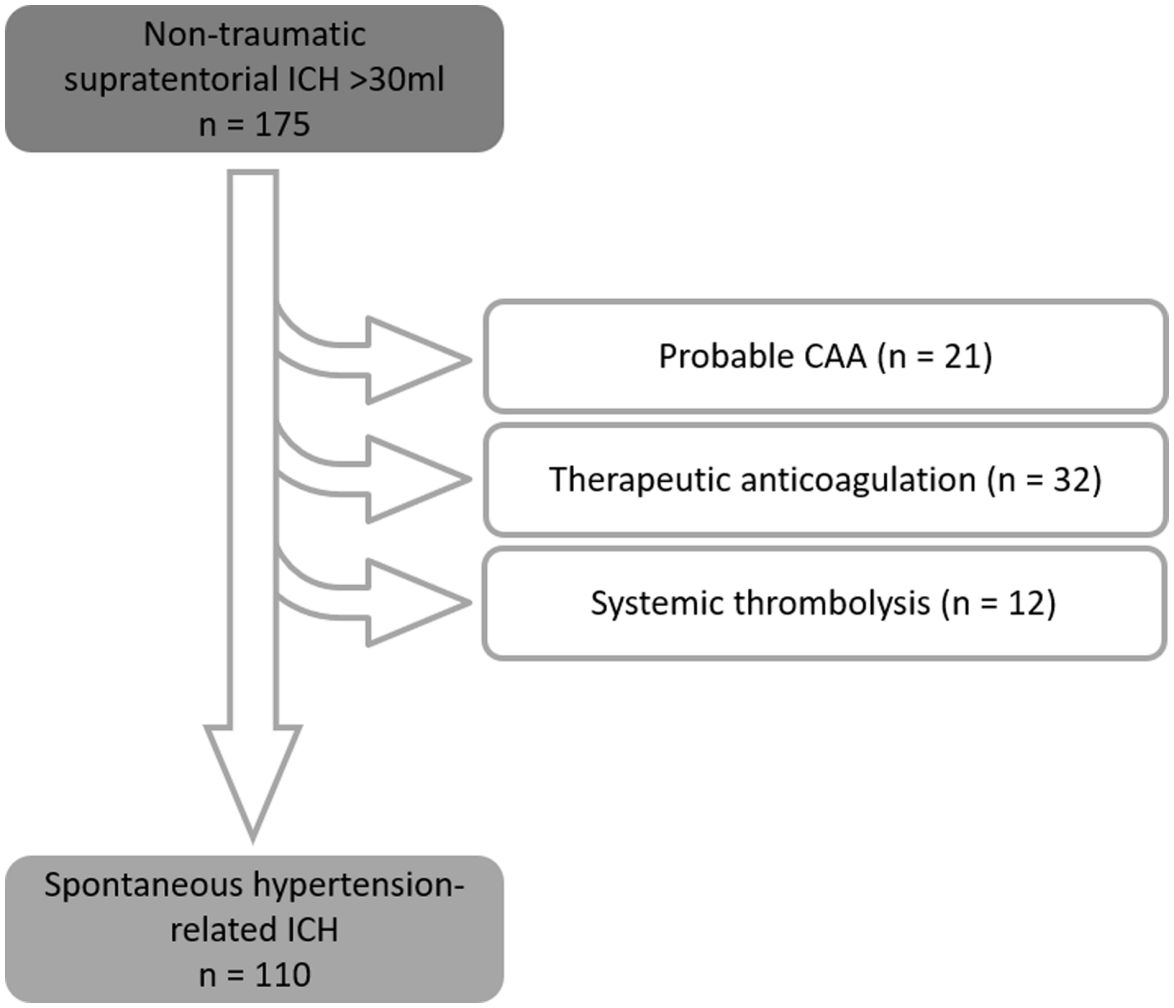
Exclusion scheme, resulting in 110 patients with spontaneous, non-traumatic supratentorial hemorrhage with an initial volume > 30 mL.

A total of 175 patients were identified with a supratentorial ICH volume of >30 mL who had been treated with bedside catheter aspiration; 65 of these were excluded from analysis due to the following specific causes of ICH: cerebral amyloid angiopathy (*n* = 21), therapeutic anticoagulation (vitamin-K antagonists, dabigatran, rivaroxaban, apixaban, edoxaban, heparin; *n* = 32), and systemic thrombolysis with recombinant tissue plasminogen activator for ischaemic stroke (*n* = 12). Thereby, 110 patients with spontaneous, non-traumatic supratentorial ICH were included in the analysis (see Figure 2). The supratentorial ICH in these patients was likely due to hypertension-related cerebral small vessel disease (92 patients with known history of hypertension, 18 without a history of hypertension, but with the absence/exclusion of other causal reasons for ICH) (Table 1).

**Table 1:** Patient characteristics (*n* = 110).

Baseline characteristics	
Age (years)	74 (64–79)
Sex (%female)	48
Hypertension (%)	83.6
Diabetes mellitus (%)	20.9
Aspirin use (%)	31.8
Statin use (%)	27.3
Haematoma volume on admission (mL)	60.6 (50.5–82.5)
Left-hemispheric ICH (%)	50.9
Lobar ICH (%)	66.4n
IVH (%)	55.5
Length of stay (days)	13 (9–18)
NIHSS on admission	18 (15–22)
NIHSS on discharge	10 (6–16)
GCS on admission	11 (9–12)
GCS on discharge	14 (13–15)
mRS on discharge	4 (3–5)
Intrahospital mortality (%)	8.2

Values are presented as median and interquartile range (in parentheses) in cases with continuous variables and as percentage in cases with categorical variables. ICH, intracerebral hemorrhage; IVH, intraventricular hemorrhage; NIHSS, national institute of health stroke scale; GCS, glasgow coma scale; mRS, modified Rankin Scale.

Median age was 74 years (IQR 64–79); 66% (73/110) of patients suffered from lobar ICH and 34% (37/110) from ganglionic ICH. Fifty-six patients had left-hemispheric and fifty-four had right-hemispheric ICH.

The median time from symptom onset to CT at admission was 3.0 h (IQR 1.5 to 4.0). Catheter placement was performed 9.0 h (IQR 7.0 to 13.4) after symptom onset.

Thrombolysis (urokinase 5,000 IE every 6 h) was performed for a median duration of 3 days (range: 1 to 4 days). In 7 patients (6%), no local thrombolysis was performed because single-step aspiration had already reduced the haematoma volume to ≤15 mL.

### 3.2. Evolution of ICH volume, PHE volume and midline-shift

#### 3.2.1. ICH

**Figure 3:**
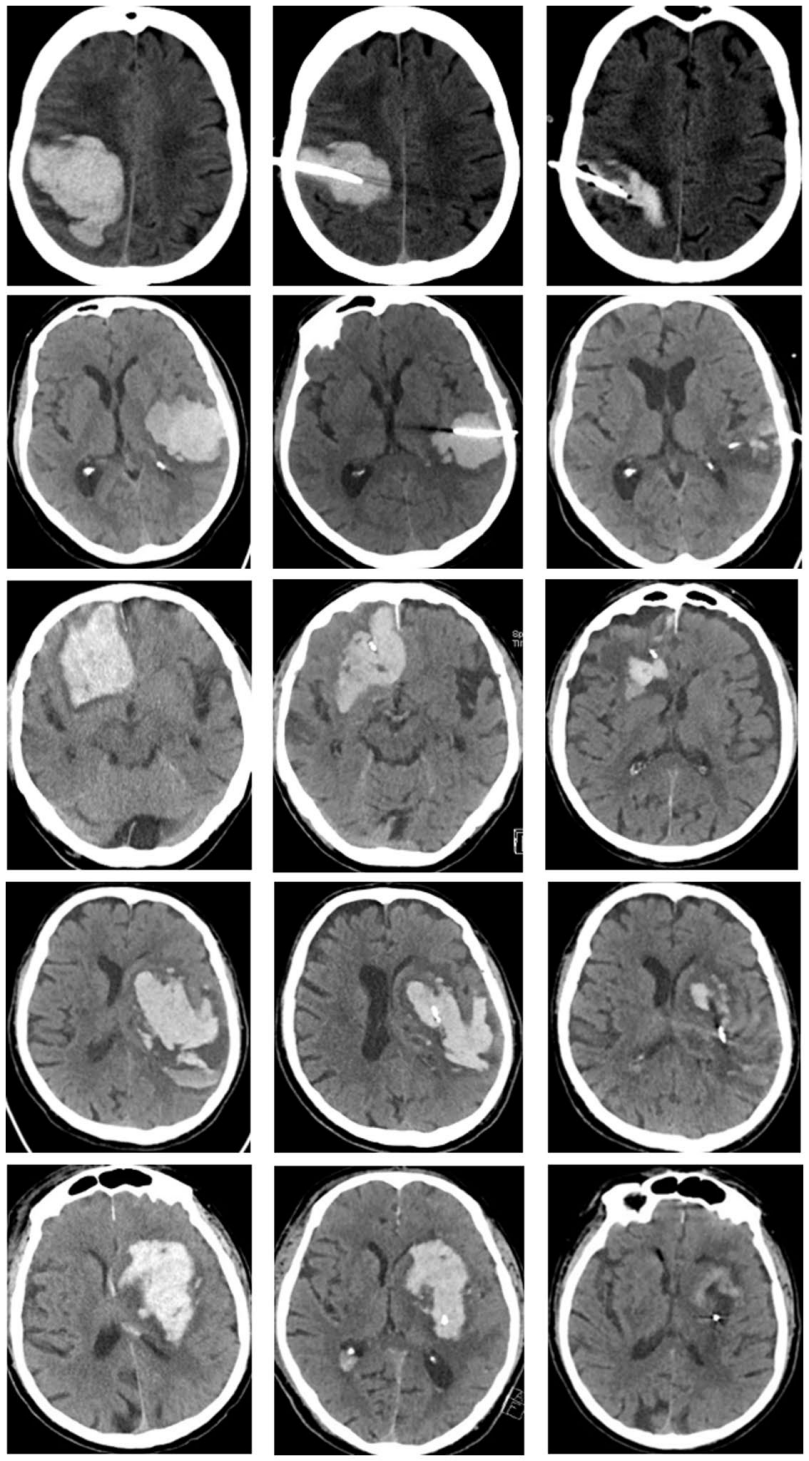
CT scans of differently located lobar ICH (row 1: occipital, row 2: temporal, row 3: frontal) and ganglionic ICH (row 4: posterior ganglionic, row 5: anterior ganglionic). CT images at admission (first column), immediately after catheter placement (second column), and at day 4 after completion of local lysis (last column).

Figure 3 shows CT scans of three differently located lobar ICH (occipital, temporal, frontal) and of a posterior and anterior ganglionic ICH with the corresponding catheter trajectory at different time points (at admission, immediately after catheter placement, and after final urokinase application on day 4).

**Figure 4:**
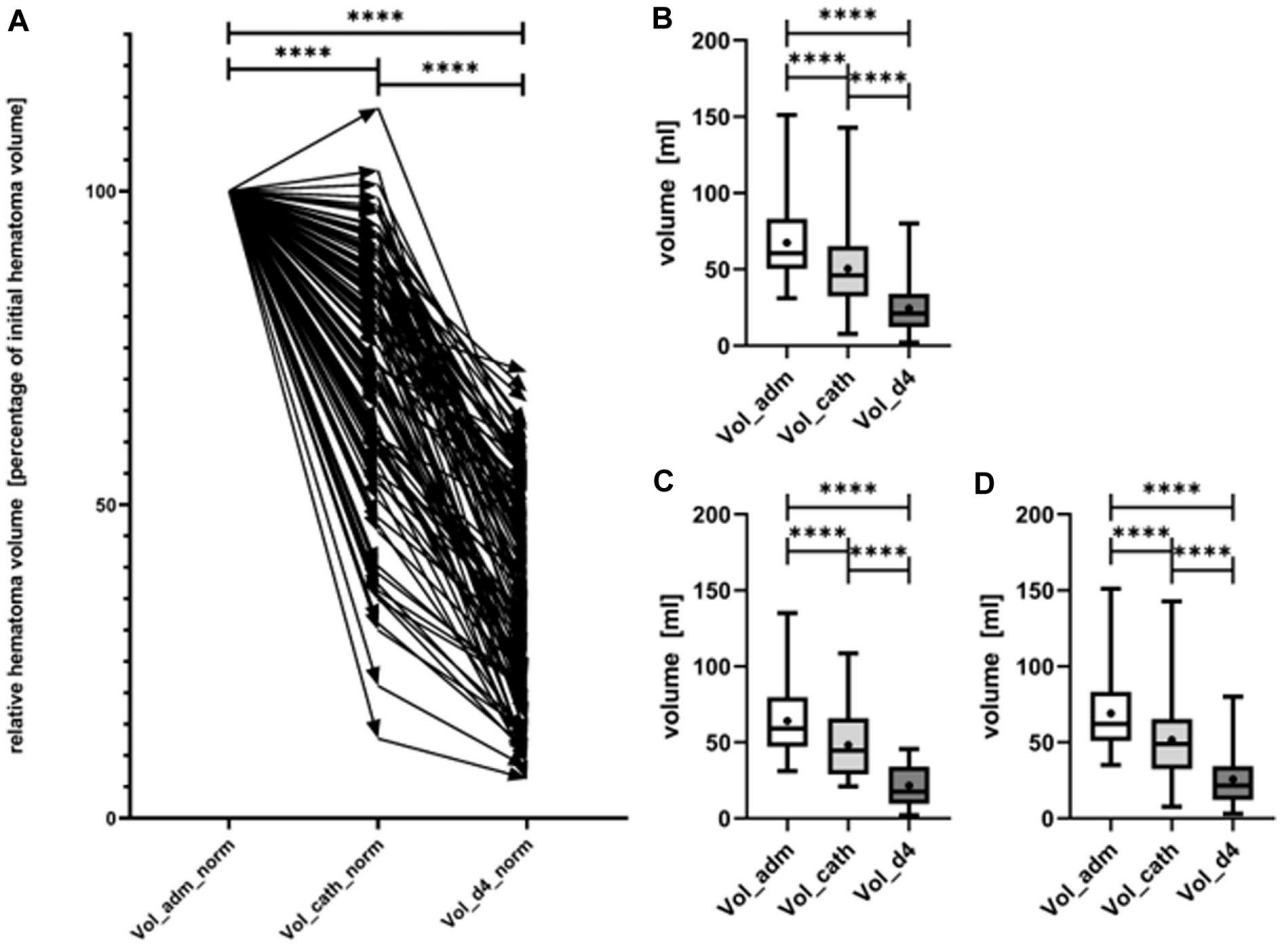
Evolution of hematoma volume from admission (Vol_adm) over the timepoint of catheter placement (Vol_cath) to day 4 after completion of local lysis (Vol_d4). In **(A)** the relative course of hematoma volume of every single patient (represented by arrow) is shown. Evolution of absolute hematoma volumes, shown as box plots with maximum and minimum displayed by whiskers is shown for all patients **(B)**, separately for patients with deep ICH **(C)**, and lobar ICH **(D)**. Hematoma volume decreased significantly from admission to immediately after catheter aspiration as well as from catheter aspiration to day 4 (*p* < 0.0001). ****symbolizing *p* < 0.0001.

The evolution of haematoma volume in all patients, as well as in the lobar and ganglionic ICH subgroups, is illustrated in Figure 4. Median haematoma volume at admission was 60.6 mL (IQR 50.5–82.5 mL), and decreased to 46.1 mL (IQR 32.3–65.0 mL) (median reduction of 21.3%, *p* < 0.0001) immediately after catheter aspiration. A further reduction to 21.0 mL (IQR 12.0–34.0 mL) was observed at the conclusion of local urokinase treatment (CT at day 4), with a median reduction in haematoma size of 67.2% compared to the initial volume (*p* < 0.0001) (Figure 4). Neither the initial haematoma volume nor the evolution of haematoma volume over time differed significantly between lobar and ganglionic ICH.

#### 3.2.2. PHE

**Figure 5:**
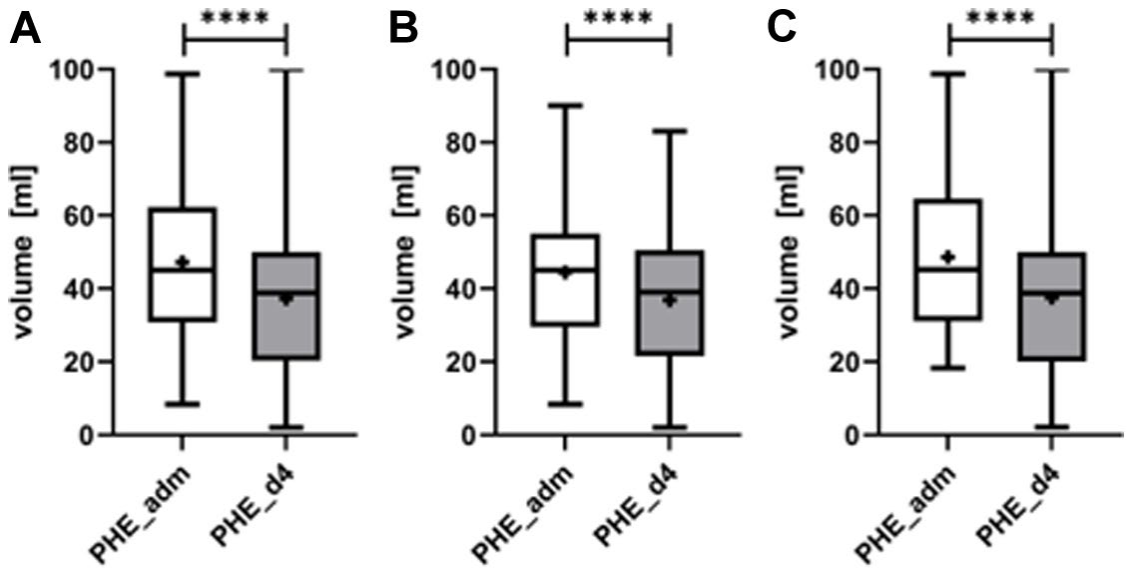
Evolution of the perihematomal edema (PHE) from admission (PHE_adm) to day 4 upon completion of local lysis (PHE_d4) for all patients **(A)**, separately for patients with deep ICH **(B)**, and lobar ICH **(C)**. PHE volume decreased significantly from admission to day 4 (*p* < 0.0001). ****symbolizing *p* < 0.0001.

Median PHE volume at admission was 45.0 mL (IQR 31.1–61.5 mL), and decreased significantly by 21.1% to 38.9 (IQR 20.6–49.8 mL) (*p* < 0.0001) on day 4 (Figure 5). Neither the initial edema volume nor edema evolution differed significantly between lobar and ganglionic ICH.

#### 3.2.3. Midline shift

The median midline shift (MLS) had already decreased significantly after catheter aspiration from 6.0 mm (IQR 4.0–9.0 mm) to 5.0 mm (IQR 3.0–7.0 mm) (*p* < 0.0001) and continued to decrease after urokinase irrigation to 2.0 mm (IQR 1.0–4.0 mm), resulting in a significant reduction in midline shift between admission and day 4 (*p* < 0.0001). The initial midline-shift was significantly more severe in ganglionic (median: 7.0 mm) compared to lobar ICH (median 6.0 mm) (*p* < 0.05). There was no significant difference in the course of midline-shift between ganglionic and lobar ICH.

**Figure 6:**
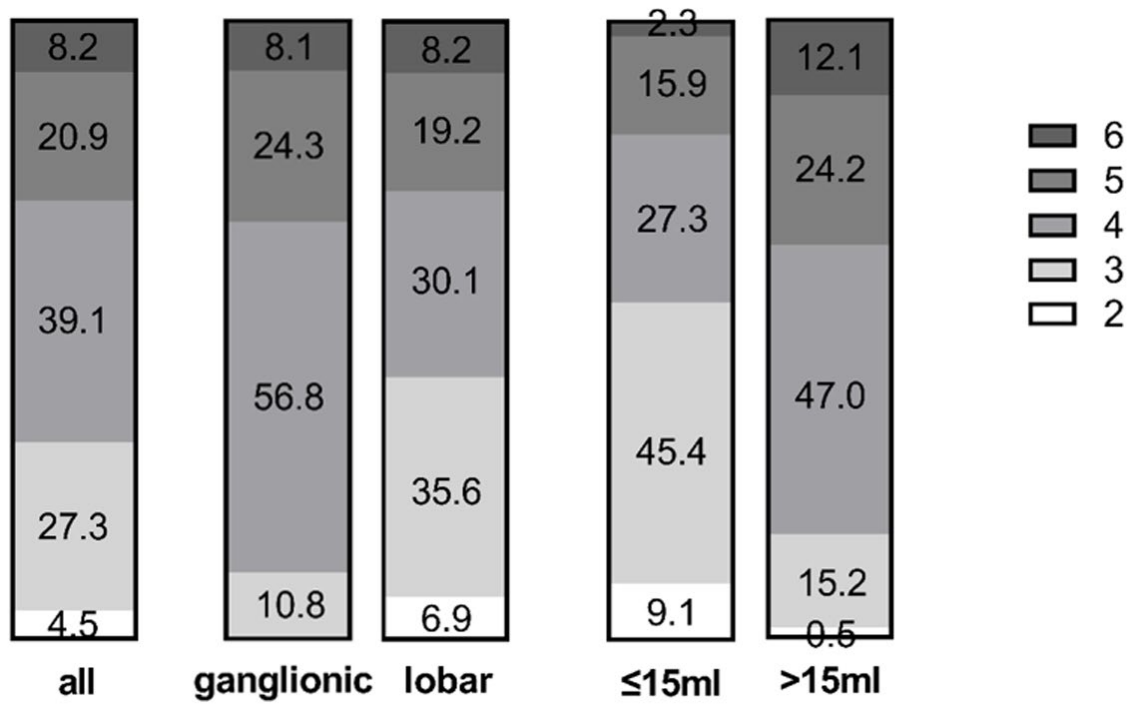
Distribution of mRS scores at discharge in the entire cohort (left) and the ganglionic vs. lobar groups (middle). The volume sub-groups (>15 mL versus ≤15 mL on day 4) are shown on the right. Numbers display the percentage in the respective group.

### 3.3. Catheter placement

According to CT scans performed after catheter placement, the catheter was in a satisfactory position in 82/110 patients (74.5%, lobar: 55/73 = 75.3%; ganglionic: 27/37 = 73.0%), defined by the fenestrated segment fully engaging with the ICH and hence being suitable for urokinase application.

In 9/110 patients (8.2%), the tip of the catheter was located outside the haematoma while the body of the catheter engaged the clot. In these cases, the catheter was retracted by a few centimeters, and the repeat control CT revealed a favourable catheter position for thrombolysis in all cases (lobar: 8/73 = 11.0%; ganglionic: 1/37 = 2.7%).

Complete catheter replacement was necessary in 19/110 patients (17.3%), since the fenestrated distal segment was insufficiently engaged with the blood clot in the control CT (lobar: 10/73 = 13.7%; ganglionic: 9/37 = 24.3%). In 2 patients (1 with lobar and 1 with ganglionic ICH), the catheter had to be replaced twice.

In general, neither position correction (replacement or retraction), nor replacement or retraction in particular, differed significantly in lobar vs. ganglionic ICH (chi-square test).

Immediate aspiration of blood upon catheter placement was possible in all patients (range 3 to 60 mL), with a median aspiration volume of 25 mL (IQR 15–35).

### 3.4. Functional outcome

The median duration of the in-hospital stay was 13 days (IQR 9–18 days).

In all patients, the median NIHSS scores improved from 18 (IQR 15–22) at admission to 10 (IQR 6–16) at discharge (*p* < 0.0001). The NIHSS scores only deteriorated in one patient (with lobar ICH) between admission and discharge.

NIHSS on admission was significantly higher in ganglionic ICH (median NIHSS: 20) compared to lobar ICH (median NIHSS: 17) (*p* < 0.01). The degree of NIHSS improvement from admission to discharge showed no significant difference between lobar and ganglionic ICH (a median of −7 and − 6.5 points, respectively), but NIHSS at discharge was still significantly higher in ganglionic compared to lobar ICH (*p* < 0.01).

The median GCS at admission was 11 (IQR 9–12) and increased to 14 (IQR 13–15) at discharge (*p* < 0.0001). GCS at admission did not differ significantly between lobar and ganglionic ICH (11 and 9 respectively). Moreover, the improvement in GCS between admission and discharge did not differ significantly between lobar and ganglionic ICH (median improvement of 3 in both groups).

The median mRS at discharge [median day 13 (9–18)] was 4 in all patients.

Thirty-two percent (35/110) of all patients had a good outcome, with an mRS of ≤3 (able to walk short distances without assistance), while 8.2% (9/110; 6 with lobar and 3 with ganglionic ICH) died. The reason for death was withdrawal of care due to: (i) very poor prognosis following a lack of improvement after catheter therapy (*n* = 6 patients at 3–4 days post-ictus) and (ii) sepsis and multiorgan failure (*n* = 3). None of the causes of death was due to an intracranial complication of ICH, such as mass effect, increased ICP or herniation.

mRS at discharge differed significantly between ganglionic and lobar ICH patients (*p* < 0.05): outcome was good (mRS ≤ 3) in 42.5% of the lobar group compared to 10.8% in the ganglionic group (Figure 6).

### 3.5. Sub-group analysis of patients with the target haematoma volume of ≤15 mL

On day 4, haematoma volume was reduced to ≤15 mL in 44 out of 110 (40.0%) patients (ganglionic 43.2% versus lobar 38.4%). In this group, 55% of patients had an mRS ≤3 at discharge, which was significantly higher than that in the group of patients who did not reach the target volume of ≤15 mL (11/66, 17%; *p* < 0.001).

This effect was mainly evident in the lobar group, where 71% of patients (20/28) had an mRS ≤ 3, compared to 25% in the ganglionic group (4/16).

The improvement in NIHSS was more pronounced in patients whose haematoma volume reached ≤15 mL (median improvement of 8 points) compared to those who did not reach this target (median reduction of 6 points); however, the difference was not significant (*p* = 0.08).

### 3.6. Complications

#### 3.6.1. Re-bleeding

Secondary or *de novo* bleeding was observed in a total of three patients, each at different time points: in one patient, an asymptomatic, 13% increase in haematoma volume was detected between the initial CT and the post-catheter placement CT. The second patient showed an asymptomatic, secondary increase in haematoma volume after 2 days of local lysis. The third patient suffered from a new haemorrhage adjacent to the initial haematoma, 60 h after the final dose of urokinase.

#### 3.6.2. Secondary open surgery

In one patient with a large right-sided haematoma (120 mL, MLS of 16 mm, NIHSS = 18), haematoma volume was reduced to 81 mL and MLS to 13 mm following catheter aspiration and local lysis for 48 h. On day 3, the NIHSS score had increased to 21; the control CT showed a progressive space-occupying PHE with a MLS of 17 mm, while haematoma volume was reduced to 71 mL. Due to the mass effect of the progressive edema, open surgery with haematoma evacuation was performed. At the time of discharge to rehab, the patient had an NIHSS score of 21.

#### 3.6.3. Infections

A total of 3 patients suffered from a catheter-associated cerebral abscess. The associated clinical signs appeared 12, 13 and 15 days after catheter removal, respectively. Symptoms included *de novo* headache and worsening of pre-existing neurological deficits (*n* = 2) and *de novo* drowsiness and disorientation in the third patient.

All three patients had initially suffered from a lobar haematoma located temporo-occipitally, with the entry point of the catheter closest to the haematoma.

Surgical evacuation was performed immediately after identification of the abscess, followed by antibiotic therapy adapted to the antibiogramm of the detected bacteria (*Staphylococcus aureus* in 2 and *Propionibacterium acnes* in 1 patient). Patient monitoring upon treatment showed no residual clinical signs of the cerebral abscess in all 3 patients, and the mRS score 8–10 weeks after the initial haemorrhage was 2, 3 and 3, respectively.

## 4. Discussion

This is the first study to describe a free-hand bedside catheter technique that is supplemented with urokinase fibrinolysis for clot evacuation in patients with large, supratentorial lobar or ganglionic spontaneous ICH.

Application of this technique resulted in an overall significant reduction in haematoma volume, PHE and MLS, all within 4 days of ictus. These imaging-based findings were associated with a reasonably good short-term outcome at discharge in lobar ICH, especially in those whose haematoma volume was able to be reduced to ≤15 mL. In ganglionic ICH short-term outcome was worse. MIS was confounded by re-bleeding in three patients only, each at different timepoints. Cerebral abscesses represented the most common complication and occurred in three patients, albeit without clinical residuum after surgical and antibiotic treatment.

### 4.1. Haematoma reduction

Haematoma volume has been found to be strongly related to poor outcome in patients with ICH (20). However, those open surgery studies, that are based on the assumption that a rapid reduction in mass effect may be beneficial, have so far failed to demonstrate that craniotomy-related clot removal is advantageous in comparison to standard medical treatment (6, 7, 21). These negative results were mainly ascribed to the occurrence of surgically-induced trauma in the surrounding brain tissue, which outweighed the benefit of haematoma evacuation. To avoid such secondary injuries, several minimally-invasive procedures have subsequently been developed, with encouraging results (4, 8, 9). Recently, the critical view on surgical interventions has been reinforced by evidence of surviving white matter fibers within the hematoma that could be damaged by surgical interventions, which in turn may negatively impact self repair mechanisms and thereby functional outcome (22, 23).

In the present study, application of single-step catheter aspiration directly after catheter placement immediately reduced haematoma volume by 22.3% of the initial volume. Subsequent local thrombolysis for a median of 3 days led to a further reduction in volume of 68.2% of the initial volume. This finding is in the upper range of previous catheter-based evacuation studies (24), and similar to the results of the MISTIE III trial, which reported a post-procedural reduction in haematoma volume by 69% in ICH patients with a volume of ≥30 mL, who had undergone stereotactic CT-guided catheter aspiration followed by rtPA application (10).

PHE was significantly reduced by 21.1% after 4 days when compared to edema at admission. This reduction is remarkable in comparison to the approximate 60% increase that is reported under standard medical care (25), and is comparable to the decrease of 17% in the MIS group reported in MISTIE II (26).

As a result of the reduced haematoma size and edema volume, MLS (as an indicator of the lesion’s mass effect) decreased immediately after catheter aspiration and continued to decline significantly until day 4 following local lysis.

### 4.2. Timing of intervention

The optimal time points for both open surgery and MIS in spontaneous ICH have not been definitively identified. Some neurosurgeons advocate postponing surgical intervention until the haematoma has stabilized; this is based on the notions that (i) haematoma expansion most frequently occurs in the hyperacute phase (27, 28), and (ii) there is an increased risk of re-bleeding and mortality following ultra-early craniotomy and clot evacuation within 4 h upon symptom onset (29). The MISTIE trials dealt with this issue by performing at least one stability CT to confirm a haematoma growth of less than 5 mL. Of note, catheter placement was performed at a mean of 58 h after ictus in the MISTIE III trial. However, based on pathophysiological evidence and animal studies, early surgery is also likely to be beneficial in terms of preventing a mass effect and an increase in ICP. Moreover, secondary brain injury resulting from the toxic effect of blood degradation products, inflammation, and edema progression, would most likely be reduced. Two recent meta-analyses also shed some light on the timing of surgical intervention: whereas one meta-analysis found similar benefits when MIS was performed within 24 and within 72 h (30), another reported a more pronounced benefit of haematoma evacuation when MIS was performed earlier, although the authors did emphasize that the average time to surgery was still >8 h in all but one of the studies analysed (11). In contrast to MISTIE, in our study, follow-up CT was performed before drainage placement only in cases of clinical deterioration. The purpose of this was to minimize the time between admission and the MIS procedure in patients had who already presented with reduced consciousness due to the mass effect of the haematoma. By omitting the routine stability CT performed in MISTIE III and applying our bedside therapy concept, catheter placement was able to be achieved within 9 h of symptom onset (and 5.8 h after admission). Thus, the median time from onset to intervention in our study corresponds to the lower range of the studies assessed by Sondag et al. (>8 h) (11). Otherwise, it lies distinctly above the “ultra-early” timing of Morgenstern et al. (<4 h) (29), but distinctly below the “early surgery” in STICH II (26 h) (7). In contrast to the reported complication of re-bleeding in ultra-early surgery within 4 h (29), we did not observe haematoma growth or re-bleeding in all but one patient (without any concomitant clinical effect) in the routine control CT immediately after catheter positioning. Therefore, the technique presented here combines the general advantages of MIS with those associated with a short delay. This is mainly due to its bedside-setting, which frees it from the additional need for extra personnel, space and equipment.

### 4.3. Functional outcome

In the MISTIE III trial, no significant difference in the proportion of patients with a good outcome (mRS ≤3) was found between the surgical and medical treatment groups at 1-year follow-up; however, patients in whom the haematoma reduction goal of ≤15 mL was reached after treatment had a 10-fold higher chance of a good outcome (10). In general, the proportion of patients with good outcome was unexpectedly high (control: 41%, MIS: 45%), especially when compared to the control group in the Phase 2 MISTIE II trial (20% with a good outcome at 6 months/ 33% with good outcome in the treatment group) (31). In the timepoint-focused STICH trials – in which just 1% of patients actually received MIS, despite all types of surgery generally being allowed – a similar proportion of patients achieved good outcomes after 6 months in the control and the treatment groups, respectively (STICH 24%/ 26% and STICH II 38%/ 41%) (6, 7).

The mortality rate after 1 year was higher in the control group compared to the treatment group (27% vs. 20% respectively) in MISTIE III (10), again distinctly differing from data of MISTIE II where 36 and 40% of patients had died after 1 year in the control and treatment group, respectively (31). Mortality in the STICH trials also did not differ significantly between control and treatment groups (37% vs. 36% in STICH, 24% vs. 18% in STICH III) (6, 7).

Meta-analyses of stereotactic/neuroendoscopic aspiration versus craniotomy found significantly reduced odds of death or dependency at the final follow-up (8, 9). Two recent meta-analyses suggested that outcome is improved in patients treated with minimally invasive procedures compared to medical treatment and craniotomy. This effect was even more pronounced when intervention was performed early after symptom onset (11, 30).

In the present study, the mRS score at discharge served as a proxy for short-term functional outcome, yielding a median score of 4. Although this outcome may seem poor, it should be taken into account that the mRS is usually used for the assessment of long-term outcome, and hence may not be sensitive enough to capture subtle changes in clinical course during the acute to sub-acute phase in patients with large ICH. However, by applying the NIHSS assessment, that appears to capture more subtle changes in the acute phase, we observed a significant neurological improvement from admission (median NIHSS: 18) to discharge (median NIHSS: 10), after a median stay of 13 days following catheter aspiration and local lysis. Hereby, 92% of patients showed neurological improvement during their in-hospital period.

Nevertheless, the short-term outcome results in the present study are comparable to the long-term outcome results of MISTIE II and STICH studies, which include both ganglionic and lobar haematoma (6, 31).

Moreover, in our previous analysis of catheter-based haematoma evacuation in CAA patients, the proportion of patients with good outcome (mRS ≤3) increased from 33% upon acute hospitalization (median 2 weeks post ictus) to 48% at the conclusion of rehabilitation (median discharge time: 9 weeks post-ictus) (14). Longitudinal data from MISTIE III showed a 4-fold increase in functional independence between day 30 and day 365 in both groups (10). Therefore, it is reasonable to assume that there was an increase in the proportion of patients with good functional outcome in our cohort over time, even if short-term outcome (<30 days) was not reported in MISTIE III, and our patient cohort was older (median age 74 years versus 62 years), had larger haematoma volumes (median volume 60.6 mL versus 41.8 mL), and 55.5% had an additional intraventricular hemorrhage (versus no patients in MISTIE III). Since the current cohort was younger (median age 74 years compared to 79 years) and had has smaller hematoma volumes at admission (median volume 61 mL versus 70 mL) compared to the previously CAA-cohort (14), we except at least a similar increase in the proportion of good outcome.

In the present study, an mRS outcome score of ≤3 at short-term (2 weeks) was observed in 32% of the entire patient group and in 44% of patients with lobar ICH. In conjunction with a mortality rate of 8.2%, this outcome appears comparatively good benchmarked against previous reports. Due to the retrospective nature of our study and the large observation period, we could not evaluate mid- or long-term outcome.

### 4.4. Deep versus lobar ICH

Although neither the initial haematoma and edema volumes nor their evolution over time differed significantly between deep and lobar ICH, short-term outcome was significantly worse in ganglionic ICH. This might be due to the direct damage of essential structures located in the ganglionic area, such as the pyramidal tract and thalamus, which leads to more severe clinical deficits in the ganglionic sub-group that is already evident at admission (NIHSS at admission significantly higher in ganglionic ICH).

The STICH trial observed potential benefits of early surgery in the superficial hemorrhage subgroup, whereby the authors suggested that the advantage of clot evacuation via craniotomy in superficial haematoma may be attributable to less risk of extensive surrounding tissue damage compared to that in deep haematoma (6). However, this could not be unambiguously confirmed by individual patient data meta-analysis (21) nor by the results of STICH II, in which superficial location was an inclusion criterion (7). Instead, a recent meta-analysis showed that haematoma evacuation with craniotomy or minimally invasive techniques had a more robust beneficial effect than medical therapy in patients with deep ICH, when compared to those with lobar ICH. The authors attributed this to the modifying effect of location, which might vary between craniotomy and minimally invasive haematoma evacuation (11). The idea of haematoma location (lobar versus deep) influencing the effects of different surgical techniques (craniotomy versus MIS) may be further supported by the fact that the MIS group in MISTIE III displayed a functional outcome that was comparable to the standard care group, despite the higher proportion of patients with lobar haematoma in the latter group (10), the location that had previously been attributed to a better prognosis (7).

Technically speaking, however, defining and accessing the entry point of the trajectory with free-hand catheter positioning appears to be easier in lobar haematoma patients than those with deep ICH. In lobar ICH, the catheter only needs to be positioned in the superficial area closest to the haematoma. However, in the present study, even though catheter placement in deep ICH is theoretically more technically challenging, this is not reflected by a more frequent need for replacement or retraction of the catheter in this cohort.

### 4.5. Complications

One major complication of free-hand catheter placement and immediate blood aspiration is an increased risk of re-bleeding. We used a 3D-reconstructed CT scan to optimize the location of both the entry point and trajectory of the catheter, depending on the exact location of haematoma. In addition, aspiration immediately after catheter placement was performed very carefully, since vigorous aspiration can result in re-rupture of injured vessels. Here, we observed a total of three cases of re-bleeding at different timepoints in relation to catheter placement and local lysis: (i) directly upon catheter placement (*n* = 1, asymptomatic), (ii) after four applications of urokinase (*n* = 1, asymptomatic), and (iii) 60 h after cessation of local lysis (*n* = 1, symptomatic). Therefore, the reported technique does not appear to be associated with a significant risk of symptomatic re-bleeding. Furthermore, the correction of catheter position (replacement or retraction), which was necessary in 25.5% of patients in our cohort, did not lead to re-bleeding. Nevertheless, since more accurate freehand catheter positioning is associated with a reduction in the need for subsequent repositioning (32), an improvement in accuracy is desirable, and could be achieved by novel approaches such as sonographic assistance (32) or augmented reality (33).

Bacterial brain infection as a complication of catheter-based local thrombolysis has been described in 1–14% of patients with supratentorial ICH (10, 34, 35). In our cohort of 110 patients, three patients suffered from cerebral abscess with physiological skin pathogens (2.7%). All of these patients had lobar temporo-occipital hemorrhage, but were treated with catheter-based local clot lysis over different time spans (24–120 h). Herewith, by reflecting the complete spectrum of duration of catheter/local lysis duration, we do not see an association between duration and infection rate. Based on the location of the haematoma, the catheter was placed at a dorsal entry point in all three patients. Thus, it could be postulated that this particular catheter location (*n* = 31 in total) was associated with a higher risk of bacterial brain infection, likely due to the continuous contact between the skin of the occiput (and hence the entry point of the catheter) and the bed. Further studies are needed to investigate this possible correlation between catheter location and risk of infection. Due to the high infection rate of 9.7% (3/31) in the subgroup of patients with a temporo-occipital entry point of the catheter a prophylactic antibiotic treatment covering the most common (skin) pathogens might display a reasonable consideration.

### 4.6. Local lysis

Local clot lysis with rtPA or urokinase has previously been described in intraventricular hemorrhage (36), isolated traumatic hematoma (37) and spontaneous (10) and anticoagulant-associated (34) hemorrhage, resulting in faster blood clot resolution, without an increase in the rate of rebleeding. Here, we used urokinase as fibrinolytic agent since it has been shown that it is at least as efficient as rtPA in local clot lysis (38–40). Additionally, the well-established neurotoxicity and pro-inflammatory effects of rtPA are not described for urokinase (39, 41). Whereas dosage of rtPA is now less controversial and thus widely set to 1 mg every 8 h, dosage of urokinase remains controversial (in intracerebral or intraventricular hemorrhage). The reported doses range from 5,000 IE to 25,000 IE per application (34, 42). Here, we used the dosage of 4 × 5,000 IE per day for a maximum of 4 days, since a higher single dose of urokinase would need a higher application volume, with the risk of increasing intracranial pressure. Furthermore, a prolonged period of catheter-based local lysis (≥5 days) may be harmful due to an increase of infection rate over time upon catheter placement (43), and further reduction of the hematoma after day 4 is usually minimal.

### 4.7. Limitations

Our study has several limitations, namely, its retrospective design and single-center setting, as well as the lack of a control group, which clearly limits generalization of our findings. The latter also precludes the possibility of making a definitive statement about the actual efficacy of this catheter-based approach compared to standard medical treatment or open surgery. However, because this study was intended as a feasibility study, it has provided the first insights into the safety and haematoma-reducing capacity of this bedside technique in large spontaneous supratentorial ICH. A large randomized-controlled trial is now necessary to confirm our preliminary observations. The feasibility of applying this new technique across a multicentre basis also needs to be investigated, especially with relation to MISTIE III, where the success of reaching a target volume of ≤15 mL correlated with the degree of site and surgeon experience (10). However, in our cohort, the long observation period of 10 years resulted in the participation of a considerably large group of surgeons with various levels of training. Moreover, because general anesthesia and an operating room are not required for this technique, transferring it to a broad range of centers or settings will be easier.

Surely, the technique is open for further improvement, for example a more specific and elaborated planning of the trajectory by using DTI to determine – and thereby sparing –the white matter tracts [as, e.g., reported by Du et al., Steineke and Barbery and Zhang et al. (44–46)] and also predict clinical outcome (47, 48). Additionally, the supplement of tractography could be used to further evaluate the presence and role of surviving white matter tracts within the hematoma that are thought to be at risk for damage by the surgical intervention (22, 23). However, the time delay to be expected as a result of performing tractography [median time to surgery reported by Zhang et al.: 21 h (46)] should not be lost sight of, since the recently reported more pronounced benefit of hematoma evacuation when MIS was performed earlier (11).

## 5. Conclusion

Bedside catheter aspiration followed by urokinase irrigation is fast and safely feasible in patients with large, spontaneous supratentorial ICH and immediately reduces the mass effect of hemorrhage and edema. However, a better mechanistic understanding of clot evolution and resulting toxicity to the surrounding brain is needed to ensure clinical benefit to patients. The results of this retrospective study warrant further controlled studies focusing on the functional outcome.

## Data availability statement

The raw data supporting the conclusions of this article will be made available by the authors, without undue reservation.

## Ethics statement

The studies involving human participants were reviewed and approved by Ethik-Kommission der Albert-Ludwigs-Universität Freiburg, Engelberger Straße 21, 79106 Freiburg. The patients/participants provided their written informed consent to participate in this study.

## Author contributions

MH, JL, AH, RR, TD, W-DN, and JB: acquisition, analysis and interpretation of data for the work, revising the manuscript, and final approval of the version to be published. MH and JB: drafting, conception, and design of the work. All authors contributed to the article and approved the submitted version.

## Funding

We acknowledge support by the Open Access Publication Fund of the University of Freiburg.

## Conflict of interest

The authors declare that the research was conducted in the absence of any commercial or financial relationships that could be construed as a potential conflict of interest.

## Publisher’s note

All claims expressed in this article are solely those of the authors and do not necessarily represent those of their affiliated organizations, or those of the publisher, the editors and the reviewers. Any product that may be evaluated in this article, or claim that may be made by its manufacturer, is not guaranteed or endorsed by the publisher.

## Footnotes

^1^ http://www.r-project.org
